# A genome-wide expression analysis identifies a network of EpCAM-induced cell cycle regulators

**DOI:** 10.1038/sj.bjc.6604725

**Published:** 2008-10-28

**Authors:** K Maaser, J Borlak

**Affiliations:** 1 Molecular Medicine and Medical Biotechnology, Fraunhofer Institute of Toxicology and Experimental Medicine, Nikolai-Fuchs-Str. 1, 30625 Hannover, Germany

**Keywords:** epithelial cell adhesion molecule (EpCAM), lung carcinoma, proliferation, DNA microarray analysis, proliferation, cell cycle

## Abstract

Expression of the epithelial cell adhesion molecule EpCAM is upregulated in a variety of carcinomas. This antigen is therefore explored in tumour diagnosis, and clinical trials have been initiated to examine EpCAM-based therapies. Notably, the possible intracellular effects and signalling pathways triggered by EpCAM-specific antibodies are unknown. Here, we show treatment of the mouse lung carcinoma cell line A2C12, of the human lung carcinoma cell line A549 and the human colorectal cell line Caco-2 with the monoclonal EpCAM antibody G8.8 to cause dose dependently an increase in cell proliferation, as determined by the MTS and the 5′-bromo-2′-deoxyuridine (BrdU) labelling assay. Furthermore, a genome-wide approach identified networks of regulated genes, most notably cell cycle regulators, upon treatment with an EpCAM-specific antibody. Indeed, changes in the expression of cell cycle regulators agreed well with the BrdU labelling data, and an analysis of differentially expressed genes revealed the processes with the strongest over-representation of modulated genes, for example, cell cycle, cell death, cellular growth and proliferation, and cancer. These data suggest that EpCAM is involved in signal transduction triggering several intracellular signalling pathways. Knowing EpCAM signalling pathways might lead to a reassessment of EpCAM-based therapies.

The epithelial cell adhesion molecule (EpCAM) was initially described as tumour-associated antigen (Koprowski *et al*, 1979). However, EpCAM was then shown to be a panepithelial marker that is enriched at the basolateral membrane (Winter *et al*, 2003b). The level of EpCAM expression has been associated with cellular differentiation, for example the germinal regions in normal colonic crypts display high levels of EpCAM expression, which decrease as cells differentiate and migrate to the top of the villi (Schiechl and Dohr, 1987).

Expression of EpCAM is known to be upregulated in a variety of carcinomas including those of the lung and colon (Went *et al*, 2006). Recent evidence is suggestive of EpCAM to serve as a cancer stem cell marker (Yamashita *et al*, 2007). The correlation between EpCAM expression and prognosis seems to be tissue specific. In breast, ovarian, and oesophageal squamous cell carcinoma, EpCAM overexpression has been correlated with poor prognosis (Spizzo *et al*, 2002, 2004, 2006; Stoecklein *et al*, 2006). In contrast, a positive impact of EpCAM overexpression on survival was found in renal cell carcinoma and the subgroup of pT2 adenocarcinomas of the lung (Seligson *et al*, 2004; Songun *et al*, 2005). On account of its high level of expression in several carcinomas, it is a candidate target for tumour diagnosis and therapy. Several clinical trials targeting EpCAM have been conducted with variable success (Baeuerle and Gires, 2007). Mouse-derived monoclonal antibodies directed to EpCAM have been successfully used for adjuvant treatment of minimal residual disease of colon carcinoma (Riethmuller *et al*, 1994, 1998). Initially, the approach appeared to improve long-term survival of patients, but a larger study could not corroborate these promising therapeutic effects (Punt *et al*, 2002). At present, there are several ongoing clinical trials using murine or human antibodies directed against EpCAM (reviewed in Armstrong and Eck, 2003; Baeuerle and Gires, 2007). *In vitro* studies demonstrated several possible mechanisms of action. Antitumoral effects have been ascribed to antibody- and complement-dependent cellular toxicity or anti-ideotypic immune response (Fagerberg *et al*, 1995; Armstrong and Eck, 2003). Therefore, targeting EpCAM for immunotherapy mainly aims to break self-tolerance towards EpCAM. However, recent studies showed that EpCAM itself can function as a signalling molecule.

Epithelial cell adhesion molecule is a membrane protein with two EGF-like repeats, followed by a cysteine-poor region and a short cytoplasmic tail (Baeuerle and Gires, 2007). It is known as a Ca^2+^-independent, homophilic cell–cell adhesion molecule. However, there is growing evidence that EpCAM plays a functional role not only in cell adhesion, but also in diverse processes such as signalling, cell migration, differentiation, and proliferation. Despite its function as a cell adhesion molecule, it has been associated with metastasis formation (Gastl *et al*, 2000; Spizzo *et al*, 2004; Varga *et al*, 2004), and silencing of EpCAM expression was shown to decrease the migration rate (Osta *et al*, 2004; Winter *et al*, 2007). Epithelial cell adhesion molecule has been shown to be involved in the abrogation of E-cadherin-mediated cell–cell interactions by disrupting the link between *α*-catenin and F-actin (Winter *et al*, 2003a). Epithelial cell adhesion molecule itself has been shown to interact through *α*-catenin with the actin cytoskeleton, thereby inducing the formation of stress fibres (Guillemot *et al*, 2001). Moreover, a direct impact of EpCAM on cell cycle control by upregulating the proto-oncogene c-myc and the cyclins A and E has been reported (Munz *et al*, 2004). Recently, it has been shown that EpCAM formed complexes with the tight-junction protein claudin-7, the variant isoform of the cell–matrix adhesion protein CD44v6, and the tetraspanin CD9, which facilitated metastasis formation (Kuhn *et al*, 2007). Thus, EpCAM signalling might depend on the microenvironment and interaction with other membrane molecules as well as on the expression level and subsequent possible oligomerisation. However, the exact mechanisms of EpCAM signalling are yet to be elucidated.

In this study, we investigated the EpCAM-mediated signalling upon crosslinking with a monoclonal rat anti-human antibody G8.8. Therefore, we used a novel mouse lung carcinoma cell line that was isolated from the lung tumour of a c-raf/c-myc double-transgenic mouse as a model to investigate EpCAM signalling. Moreover, we compared the results obtained from this cell model with the two human cell lines A549 (lung carcinoma) and Caco-2 (colorectal carcinoma), both of which are well known to express EpCAM highly, with the goal of understanding EpCAM signalling across a broader scope of different tumour origins.

## Materials and methods

### Cell culture

The spontaneously transformed mouse lung carcinoma cell line A2C12 was isolated from lung tumours of double c-myc and c-raf transgenic mice. Cells were isolated from lung tumours of 8-month-old transgenic mice using the protocol for the isolation and culture of rodent primary respiratory cells (Hansen *et al*, 2006). The human colorectal adenocarcinoma cell line Caco-2, the human lung carcinoma cell line A549, and the mouse cell line A2C12 were cultured in Dulbecco's modified Eagle's medium supplemented with 10% foetal calf serum, 2 mM L-glutamine, 100 U ml^−1^ streptomycin, and 100 *μ*g ml^−1^ penicillin in a humidified atmosphere containing 5% CO_2_ at 37°C.

### Western blot analyses

Whole-cell extracts were prepared by harvesting and lysing the cells with lysis buffer (50 mM Tris (pH 6.8), 1.5% w v^−1^ sodium dodecyl sulphate). Samples were boiled for 10 min, sonicated 20 times at an interval setting of 0.5 (UP 200 s; Dr Hielscher, Teltow, Germany) in 500 *μ*l lysis buffer on ice, and centrifuged at 12 000 **g** for 10 min at room temperature. The supernatant was recovered. Protein content of the lysate was determined by Smith protein assay.

Eighty micrograms of total protein extracts were separated on a 12% SDS–polyacrylamide gel and blotted onto a PVDF membrane in 25 mM Tris and 190 mM glycin at 4°C for 2 h at 350 mA. Blots were blocked in Rotiblock (Roth, Karlsruhe, Germany) for 1 h and then incubated overnight at 4°C with rabbit anti-human antibodies Raf-1 (C-12), c-Myc (N-262), CD44 (N-18), or CD133 (K-18) (all of which are also recommended to detect mouse antigens respectively; 1 *μ*g ml^−1^; Santa Cruz, Heidelberg, Germany), or rat anti-mouse EpCAM antibody (2 *μ*g ml^−1^; kindly provided by Micromed, Munich, Germany). Results for CD133 expression in mouse A2C12 were confirmed using rat anti-mouse CD133 antibody (13A4, 2.5 *μ*g ml^−1^; ebioscience, San Diego, CA, USA), with protein lysate of mouse kidney as a positive control. After washing with Tris-buffered saline (25 mM Tris and 135 mM NaCl; pH 7.6) containing 0.1% Tween and incubation with horseradish peroxidase-coupled anti-IgG antibody (1 : 10 000; Chemicon, Temecula, CA, USA) at room temperature for 1 h, the blot was washed extensively, developed using enhanced chemiluminescent detection (PerkinElmer, Jügesheim, Germany), and recorded with Kodak IS 440 CF (Kodak; Biostep GmbH, Jahnsdorf, Germany).

### Flow cytometry

Cells were trypsinised, washed twice with phosphate-buffered saline (PBS; 140 mM NaCl, 10 mM Na_2_HPO_4_, 2.6 mM KCl, 1.4 mM KH_2_PO_4_ (pH 7.4)), and immunoassayed, as described previously (Maaser *et al*, 2001). Cells were incubated for 1 h at room temperature with primary rabbit anti-mouse CD44 or rabbit anti-human CD133 (both 4 *μ*g ml^−1^; Santa Cruz), or with rat anti-mouse EpCAM antibody (10 *μ*g ml^−1^; kindly provided by Micromed), respectively. Results of EpCAM expression in human cell lines were confirmed using mouse anti-human EpCAM antibody (10 *μ*g ml^−1^, clone 0.N.276; Santa Cruz). Cells were washed twice with PBS and then incubated with 4 *μ*g ml^−1^ secondary Alexa™ 488-labelled goat anti-rabbit (CD44 and CD133) or goat anti-rat (EpCAM) IgG antibody (Molecular Probes, Eugene, OR, USA), respectively, for 1 h at room temperature. Donkey anti-mouse IgG (4 *μ*g ml^−1^; Dianova, Hamburg, Germany) was used as secondary antibody for the mouse anti-human EpCAM antibody. Fluorescence was detected by flow cytometry on a FACSscan (Becton Dickinson, Heidelberg, Germany) and analysed using CellQuest software.

### Cell proliferation

Cells were plated in 96-well microtitre plates at a density of 5000 cells per well 24 h before treatment. Cells were treated with monoclonal rat anti-mouse EpCAM antibody at concentrations of 1–100 *μ*g ml^−1^ for 48 h. Cell proliferation was measured using two different methods: incorporation of 5′-bromo-2′-deoxyuridine (BrdU) and metabolic conversion of 3-(4,5-dimethylthiazol-2-yl)-5-(3-carboxymethoxyphenyl)-2-(4-sulphophenyl)-2*H*-tetrazolium (MTS).

#### Incorporation of BrdU

5′-Bromo-2′-deoxyuridine incorporation was measured using BrdU Cell Proliferation Assay (Merck, Darmstadt, Germany) according to the manufacturer's instructions. Cells were labelled with BrdU (1 : 100) for the last 4 h of incubation. Cells were washed, fixated, and incubated with mouse anti-BrdU antibody (1 : 100; 100 *μ*l per well) for 1 h at room temperature. Antibody labelling was detected by secondary peroxidase-coupled goat anti-mouse antibody (1 : 1000; 100 *μ*l per well; 30 min at room temperature). After washing, peroxidase substrate was added for 15 min. The peroxidase reaction was stopped by adding 100 ml 2.5 N sulphuric acid, and absorbance was measured using dual wavelengths 450 and 595 nm.

#### MTS assay

The CellTiter 96® AQ_ueous_ Non-Radioactive Cell Proliferation Assay (Promega, Mannheim, Germany) was used to determine the number of viable cells in culture. The MTS assay is based on the ability of viable cells to convert a soluble tetrazolium salt to a formazan product. After exposure to rat anti-mouse EpCAM antibody, MTS reagent was added and cell cultures were incubated at 37°C for 1 h. Absorbance was recorded at 492 nm (Victor multireader; PerkinElmer).

### Isolation of RNA, production of c-RNA, array hybridisation, and scanning

The c-RNA samples were prepared following the Affymetrix Gene Chip® Expression Analysis Technical Manual (Santa Clara, CA, USA). Ten micrograms of biotinylated fragmented cRNA were hybridised onto the Mouse Genome 430A 2.0 Array or the Human Genome U133 Plus 2.0 Array, respectively. The procedures for isolation of RNA, production of c-RNA, array hybridisation, and scanning were performed according to Affymetrix's manual and as described earlier (Borlak *et al*, 2005). Each hybridisation image was scaled to probe set intensity of 250 for Mouse Genome 430A 2.0 Arrays and of 200 for the Human Genome U133 Plus 2.0 Arrays for comparison between chips.

### Gene expression data analysis

Data analysis was performed as described earlier (Borlak *et al*, 2005). For single arrays, a statistical expression algorithm within the Affymetrix® Microarray Suite software (version 5) yielded gene expression values as numeric values (signal intensities) and detection calls (‘present’ or ‘absent’) produced with different algorithms. A comparison between two arrays (tumour and control lung) resulted in the signal logarithm ratio (log2 ratio) and a change call (‘increase’ or ‘decrease’) for the expression level of each gene. The group of differentially expressed genes was restricted to those genes that were detected (‘present’ call) in antibody-treated samples for the upregulated genes and in all control samples for the downregulated genes. Further criteria were a minimum intensity of 100 in treated samples, a fold change >2, and 100% of ‘increase’ calls in comparative ranking analysis for treated *vs* untreated samples for upregulated genes, and a minimum intensity of 100 in control samples, a fold change <−2, and 100% of ‘decrease’ calls accordingly for downregulated genes. The list of differentially expressed genes was submitted to Ingenuity Pathway Analysis 5.5 (www.ingenuity.com) to analyse gene functions. The significance of functional enrichment was computed by a Fisher's exact test and represented by a range of *P*-values associated with specific functions.

### Reverse transcription and real-time semiquantitative PCR

Total RNA from each sample (2 *μ*g) was used for reverse transcription (Omniscript Reverse Transcriptase; Qiagen GmbH, Hilden, Germany). Real-time PCR was performed in a mixture containing a c-DNA equivalent to 25 ng of total RNA, 1 *μ*M of each primer, 0.5 mM dNTP mixture, 0.625 U Thermostart-Taq (ABgene, Hamburg, Germany), and 1 × PCR buffer (ABgene), 0.66 mg ml^−1^ bovine serum albumin, 1.75 mM MgCl_2_, and 5% SYBR Green (Roche Diagnostics, Mannheim, Germany) in a total volume of 20 *μ*l. Real-time RT–PCR measurement was performed with the LightCycler® (Roche Diagnostics, Mannheim, Germany). Experimental conditions and detailed oligonucleotide sequence information are given in Supplementary Table S1. Specificity of primers was confirmed by agarose-gel electrophoresis of PCR products. Differences in gene expression are shown as fold changes of antibody-treated probes *vs* untreated probes and calculated by ΔΔ*C*_t_ values. Mean expression values of the mitochondrially encoded ATP synthase 6 (MT-ATP6) and glyceraldehyde-3-phosphate dehydrogenase (GAPDH) were taken as housekeeping genes.

## Results

### Expression of c-raf, c-myc, EpCAM, CD133, and CD44

**Figure 1:**
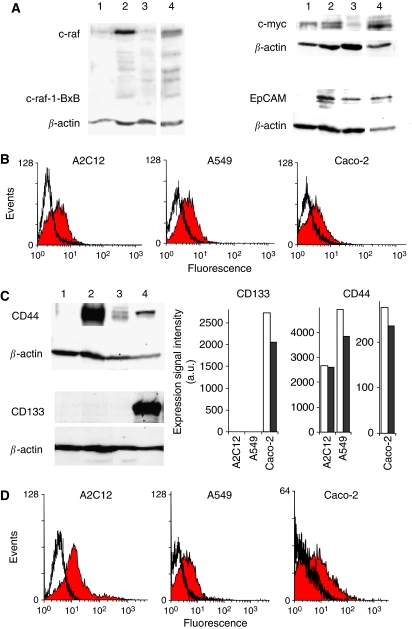
Expression of c-raf, c-myc, EpCAM, CD44, and CD133. (**A**) Expression of c-raf in A2C12 cells (lane 2) was markedly stronger than that in non-neoplastic lung tissue (lane 1), A549 cells (lane 3), or Caco-2 cells (lane 4). Expression of c-myc was detected in non-neoplastic lung tissue (lane 1) as well as in A2C12 cells (lane 2), A549 cells (lane 3), and Caco-2 cells (lane 4). Epithelial cell adhesion molecule was found to be expressed in the cancer cell lines A2C12 (lane 2), A549 (lane 3), and Caco-2 (lane 4), but not in non-cancerous lung tissue (lane 1) as determined by western blot analysis. (**B**) Epithelial cell adhesion molecule expression in A2C12, A549, and Caco-2 cells was determined by flow cytometry using anti-EpCAM antibody G8.8. The mean fluorescence of EpCAM-labelled cells (filled curve) was increased 1.98-fold for A2C12 cells, 1.92-fold for A549 cells, and 1.46-fold for Caco-2 cells as compared with the respective controls (black line). (**C**) CD44 was found to be highly expressed in the mouse lung carcinoma cell line A2C12 (lane 2), but not in non-cancerous lung tissue (lane 1), as determined by western blot analysis. A low CD44 expression was found in the cell lines A549 (lane 3) and Caco-2 (lane 4). CD133 was found to be expressed in Caco-2 cells (lane 4) only, but not in non-cancerous lung tissue (lane 1), A1C12 cells (lane 2), and A549 cells (lane 3). Expression signal intensities for CD133 and CD44 of control cells (white columns) and G8.8 antibody-treated cells (black columns), as determined by microarray analysis. In the case of more than one probe set per gene (CD44), highest intensities of the probe sets, which are specific for only one transcript designated as number_at, are shown. Signal intensities of probe sets 1419700_a_at (mouse CD133), 204304_s_at (human CD133), 1423760_at (mouse CD44), and 212063_at (human CD44) are shown. (**D**) CD44 expression was confirmed for A2C12, A549, and Caco-2 cells by flow cytometry. Cells were stained with the rat anti-human CD44 antibody (filled curve) or isotypic control antibody (black line) and secondary FITC-labelled anti-rat antibody. Results of one representative experiment each are shown. EpCAM=epithelial cell adhesion molecule.

The cell line A2C12 was isolated from lung carcinomas of mice that were double transgenic for c-myc and c-raf-1-BxB. Expression of c-myc, c-raf, and transgenic c-raf-1BxB was detected by immunoblot. The expression of wild-type c-raf (72 kDa) in A2C12 cells was markedly stronger than in non-neoplastic lung tissues, A549 cells, or Caco-2 cells (Figure 1A). However, the transgenic expression of c-raf-1BxB (42 kDa) could not be detected in these cells, indicating that A2C12 cells lost the transgenic c-raf-1BxB. Expression of c-myc was detectable in all cell lines as well as in non-neoplastic lung tissue.

All investigated cancer cell lines expressed EpCAM, as revealed by western blot analysis. In contrast, no EpCAM expression was found in non-cancerous lung tissue (Figure 1A). Expression of EpCAM in the cancer cell lines was confirmed by flow cytometry (Figure 1B).

Epithelial cell adhesion molecule was found to be highly elevated in pre-malignant hepatic tissues (Kim *et al*, 2004) and was discussed as a marker for tumour progenitor cells (Yamashita *et al*, 2007). Therefore, we investigated whether the lung and colorectal carcinoma cells used in this study express additional tumour stem cell markers such as CD44 and CD133, as we showed that they expressed EpCAM.

Expression of CD44 was shown for all investigated carcinoma cell lines, but not in healthy mouse lung tissue, as determined by western blot analysis (Figure 1C). CD44 expression was higher in the mouse lung carcinoma cell line A2C12, which was isolated from c-myc and c-raf transgenic mice, than in the human lung carcinoma cell line A549 and the human colorectal carcinoma cell line Caco-2. In contrast, the tumour stem cell marker CD133 was detected in Caco-2 cells only. mRNA expression analysis for CD133 and CD44 by microarray analysis resembled the protein expression results. No CD133 mRNA expression (detection call ‘absent’) could be detected in the lung carcinoma cell lines A2C12 and A549, whereas a high-expression signal intensity was seen in Caco-2 cells (Figure 1C). CD44 mRNA was detected in all the three cell lines. Expression of CD44 protein was confirmed by flow cytometry (Figure 1D).

### Monoclonal anti-EpCAM antibody G8.8-induced proliferation

**Figure 2:**
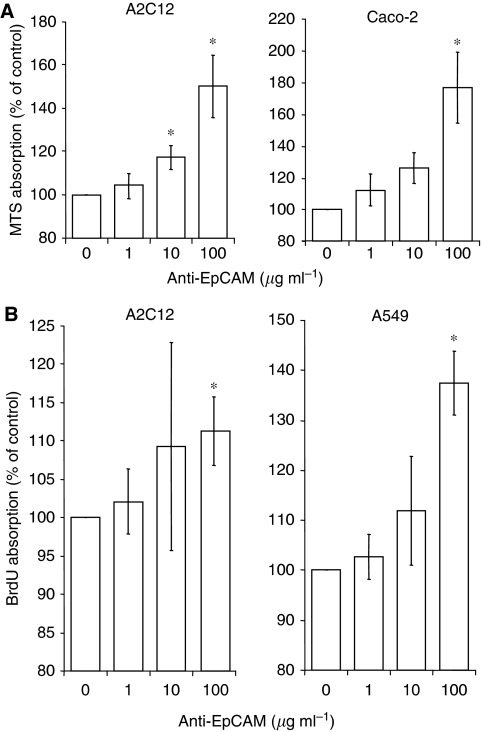
Anti-EpCAM antibody-induced proliferation. Cells were incubated with monoclonal rat anti-mouse EpCAM antibody G8.8 for 48 h, and metabolically active cells were measured using MTS assay (**A**) and proliferation was determined by measurement of incorporation of BrdU (**B**). Means±s.d. of four independent experiments is shown. ^*^P<0.05. BrdU=5′-bromo-2′-deoxyuridine; EpCAM=epithelial cell adhesion molecule; MTS=3-(4,5-dimethylthiazol-2-yl)-5-(3-carboxymethoxyphenyl)-2-(4-sulphophenyl)-2*H*-tetrazolium.

After 48 h of treatment, monoclonal rat anti-mouse EpCAM antibody G8.8 dose-dependently increased the number of metabolically active cells. A significant increase in cell number was observed in the mouse lung carcinoma cell line A2C12 already at 10 *μ*M antibody treatment and in the human colorectal carcinoma cell line Caco-2 at 100 *μ*M antibody treatment (Figure 2A). In the human lung carcinoma cell line A549, anti-EpCAM antibody induced a slight (at 10 *μ*M up to 114% as compared with untreated control), but not significant, increase (data not shown).

Next, BrdU incorporation was measured to assess the proliferation-modulating effects of the anti-EpCAM antibody G8.8. In A2C12 cells as well as in A549 cells, a significant increase in BrdU incorporation was detected upon treatment with 100 *μ*M anti-EpCAM for 48 h (Figure 2B). However, in contrast to the proliferation-inducing effects measured by MTS assay, no changes in BrdU incorporation could be measured in Caco-2 cells. This might be due to the long generation time of about 62 h of Caco-2 cells, leading to a lower sensitivity of the BrdU assay in these cells.

### Anti-EpCAM antibody-induced modulation of gene expression

The genome-wide expression profile of EpCAM antibody-treated cells was analysed to investigate the molecules involved in EpCAM signalling. Epithelial cell adhesion molecule antibody modulated gene expression in all investigated cell lines, however, to different extents (Supplementary Table S2). Gene expression modulation was most prominent in mouse and human lung carcinoma cell lines, A2C12 and A549, in both of which expression of more genes was downregulated than upregulated. In A2C12 cells, anti-EpCAM antibody treatment modulated the expression of 1973 gene transcripts (probe sets), 901 (46%) of which were increased and 1072 (54%) decreased. The modulation of gene transcription was similarly high in A549 cells (1743 modulated gene transcripts, 598 (34%) of which were increased and 1154 (66%) decreased). In the colon adenocarcinoma cell line Caco-2, less genes were modulated in total, with a higher extent of upregulation than downregulation. In Caco-2 cells, the expression of 378 gene transcripts was changed, 246 (65%) of which were increased and 142 (35%) decreased.

Analysis of function of differentially expressed genes was computed using Ingenuity Pathway Analysis software. Interestingly, the processes with the strongest over-representation of differentially expressed genes were the same for A1C12, A549, and Caco-2 cells. These processes include cell death, cell signalling, cellular growth and proliferation, cancer, cell cycle, and gene expression (Table 1A).

We further analysed genes related to cell cycle modulation with respect to their relevance for the distinct phases of the cell cycle. Most differentially expressed genes are related to the G1/G0 phase of the cell cycle in all investigated cell lines (Table 1B). It is worth noting that not all differentially expressed genes associated with cell cycle functions can be explicitly related to specific phases. Moreover, some genes were related to more than one cell cycle phase.

Three genes, which can be associated with specific cell cycle phases, were found to be commonly upregulated in all the three investigated cell lines (see also Table 2): the growth arrest and DNA-damage-inducible gene, *β* (GADD45B, all cell cycle phases), the large tumour suppressor, homologue 2 (LATS2, G1/S-phase transition and G2/M phases), and the pim-1 oncogene (PIM1, G2/M phases).

The expression of 11 different genes, which can be associated with specific cell cycle phases, was commonly modulated by antibody treatment in both lung carcinoma cell lines A2C12 and A549, but not in the colorectal carcinoma cell line Caco-2. Eight of these genes were found to be upregulated, whereas only three genes were downregulated. Interestingly, the downregulated genes include only those genes that have been exclusively associated with the G1/G0 phase of the cell cycle. These data indicate that cells in the G1/G0 phase are under-represented upon EpCAM antibody treatment, thereby reflecting the pro-proliferative effects of this treatment. Downregulated genes include CD44, met proto-oncogene (MET), and the apartyl-t-RNA synthetase (DARS). DARS is known to decrease G1/S-phase transition (Yamashita *et al*, 1999), and its downregulation by EpCAM antibody treatment might therefore contribute to the proliferative effects of antibody treatment. However, two genes exclusively linked to the G1/G0 phase were found to be upregulated in A2C12 and A549 cells, the cysteine-rich angiogenic inducer 61 (CYR61) and the dual-specificity phosphatase 1 (DUSP1). Other cell cycle-associated genes upregulated in A2C12 and A549 cells include the superoxide dismutase 2 (SOD2, G1/G0 and S phases), basic helix–loop–helix domain containing class B 2 (BHLHB2, S phase), jun B proto-oncogene (JUNB, S and G2/M phases), jun oncogene (JUN, G1/G0 and G2/M phases), leukaemia inhibitory factor (LIF, G1/G0 and G2/M phases), and MDM2 (all cell cycle phases).

Three genes, which can be associated with specific cell cycle phases, were found to be commonly modulated in both human carcinoma cell lines A549 and Caco-2. The DNA-damage-inducible transcript 3 (DDIT3), also known as GADD153, and the growth differentiation factor 15 (GDF15), which have been associated with the G1 phase of the cell cycle, were found to be upregulated. The expression of thymidylate synthetase (TYMS, G1 and S phases) was downregulated in A2C12 and A549 cells by EpCAM antibody treatment.

**Figure 3:**
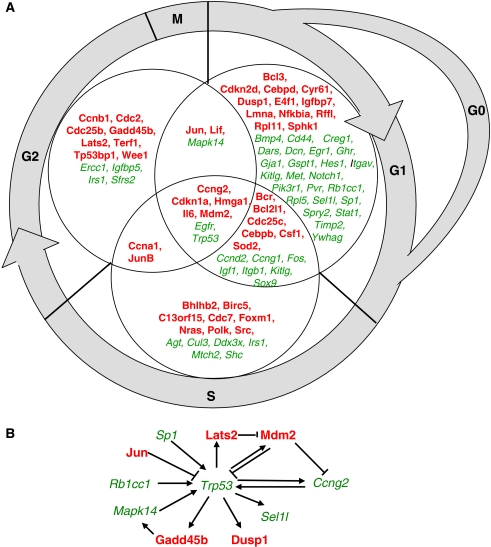
Cell cycle-related genes significantly regulated by anti-EpCAM treatment of A2C12 cells. The gene expression profile was investigated by microarray analysis, and significantly modulated genes were analysed using ingenuity software. (**A**) Shown are all the genes identified as functionally relevant to cell cycle regulation in the specific cell cycle phases. (**B**) Differentially expressed genes upon EpCAM antibody treatment of A2C12 cells, which can be considered as p53 network. Genes induced by anti-EpCAM treatment are indicated in bold, whereas genes that were repressed are indicated in italic. EpCAM=epithelial cell adhesion molecule.

Most differentially expressed genes associated with cell cycle were related to specific cell cycle phases in A2C12 cells. Therefore, we focused on A2C12 cells during single-gene analysis (Figure 3A). We identified mainly three functional groups of genes associated with cell cycle regulation: first, genes whose products are directly involved in cell cycle regulation such as cyclins, cyclin-dependent kinases, phosphatases, and cell cycle inhibitors; second, genes that code for growth factors, growth factor receptors, and their adaptor and signalling proteins. Moreover, several transcription factors were found to be differentially expressed. Several of these genes can be considered as p53 network (Figure 3B), which might play a role in the signal transduction leading to the proliferative effects of EpCAM antibody treatment. Epithelial cell adhesion molecule antibody altered proliferation similarly in the three investigated cell lines. We therefore focused on genes that were significantly modulated in A2C12, A549, and Caco-2 cells in parallel to further investigation of EpCAM signalling (Table 2). Interestingly, five out of 13 commonly modulated genes were related to cell cycle-regulating or apoptotic functions. Expression of the genes positively regulating the cell cycle (LATS2, FOSL2, and PIM1) or inhibiting apoptosis (GADD45 and PIM1) was increased, whereas the expression of a pro-apoptotic gene transcript was decreased (DIDO1). Other genes differently regulated by anti-EpCAM antibody treatment include genes for three enzymes of biosyntheses (FDFT1, LCMT2, and MAT2A), one topoisomerase (TOP2A), one gene associated with protein folding (DNAJA1), and four transcripts of unknown functions (Table 2).

### Validation of microarray data by real-time PCR

Expression of selected genes was investigated with real-time PCR using the LightCycler. Comparisons of fold changes determined by microarray analysis and real-time PCR are shown in Table 3 and Supplementary Figure S1. In almost all cases, the significant change found by microarray analysis could be confirmed by real-time PCR analysis. Although the level of altered expression varied, there was strong concordance between both methods. Only a few divergent results between the two methods have been detected, which might be due to the different probe sets/fragments of the specific gene targeted by the respective method. For example, a decreased expression of Trp53 in microarray analysis was measured only in two probe sets that recognise multiple alternative transcripts from the same gene each, whereas a third definite probe set did not recognise any modulation of expression. In general, criteria defining differential expression (see Materials and Methods) are more restricted in microarray data analysis than in real-time PCR analysis, resulting in fewer genes identified as differentially expressed by microarray analysis.

## Discussion

Epithelial cell adhesion molecule is one of the first tumour-associated antigens identified with monoclonal antibodies (Herlyn *et al*, 1979). A variety of cancer therapy strategies and clinical trials have since made use of EpCAM as a target molecule for (single-chain) monoclonal and bispecific antibodies. However, only recently has it become apparent that EpCAM itself triggers intracellular signalling. Consequently, little is known so far about the possible intracellular signalling induced by EpCAM-specific antibodies. In this study, it was shown that treatment with monoclonal rat anti-mouse EpCAM antibody G8.8 induced proliferation of mouse and human lung carcinoma cells as well as human colorectal carcinoma cells. The *in vitro* pro-proliferative effects of anti-EpCAM treatment shown in this study are surprising, as *in vivo* application of anti-EpCAM antibody resulted in tumour growth suppression (Naundorf *et al*, 2002). However, proliferative effects have also previously been shown for the mouse anti-rat EpCAM monoclonal antibody D5.7 (Wurfel *et al*, 1999). Immobilised D5.7 induced proliferation in non/low-metastasising rat pancreatic adenocarcinoma, fibrosarcoma, and pheochromocytoma cell lines, which had previously been transfected with EpCAM. These data suggest that crosslinking of EpCAM by bivalent antibodies and possible subsequent di-/oligomerisation triggers pro-proliferative signals. High EpCAM expression has been associated with an increased proliferation of keratinocytes (Schon *et al*, 1994). On the other hand, knockdown of EpCAM by EpCAM short-interfering RNA resulted in a decrease in the cell proliferation rate in four different breast cancer cell lines (Osta *et al*, 2004). Thus, both treatment with EpCAM-specific antibodies and EpCAM overexpression led to an increased cell proliferation. It is tempting to speculate that spatial proximity of EpCAM molecules, either induced by crosslinking or by higher density, may be the trigger for proliferative signalling and that both processes involve similar pathways.

The exact pathways by which EpCAM modulates proliferation are, however, unknown. In this study, we performed gene expression analysis to further identify the molecules possibly involved in EpCAM antibody-induced signalling. The number of differentially expressed genes upon EpCAM treatment differed between the cell lines. The expression of most genes was found to be regulated in A2C12 and A549 cells, less in Caco-2 cells. In this study, all cell lines were treated with EpCAM-specific antibodies for the same time period. However, the investigated cell lines differ in their biological properties, for example EpCAM expression (see Figure 2) and generation time. The doubling time of the investigated cell lines ranged from 6.7 h for A2C12 and 22 h for A549 to 62 h for Caco-2 cells. Therefore, it cannot be excluded that the quantity of differentially expressed genes depends on the biological features of the cells and adapted treatment modalities, so that for example, different antibody concentrations or different incubation times might lead to similar numbers of regulated genes.

Despite the differences in the quantity of differential gene expression, a wide overlap was detected when analysing the functions of regulated genes. Grouping the differentially regulated genes into functional processes, the cellular processes with the strongest over-representation of differentially expressed genes were the same for A1C12, A549, and Caco-2 cells. These processes include cell cycle, cell death, cellular growth and proliferation, and cancer. The identified functional groups resemble well the proliferative phenotype of cells treated with EpCAM antibody.

On the individual gene level, we concentrated on those genes that were commonly expressed in the cell lines A2C12, A549, and Caco-2. Five out of 13 commonly modulated genes were related to cell cycle-regulating or apoptotic functions. Gene expression analysis revealed that induction of proliferation was accompanied by an induction of genes whose products induce cell cycle progression (LATS2, FOSL2, and PIM1) or exert an antiapoptotic action (GADD45 and PIM1), whereas expression of pro-apoptotic genes was repressed (DIDO1). Lats2 was shown to interact physically with Mdm2, thereby inhibiting p53 ubiquitination and promoting p53 activation (Aylon *et al*, 2006). FOSL2 belongs to a family of transcription factors that have been implicated as regulators of cell proliferation, differentiation, and transformation (Tulchinsky, 2000). The GADD45B belongs to a group of genes that respond to environmental stresses and are involved in the regulation of growth and apoptosis. They are known to exhibit pro-apoptotic functions (Takekawa and Saito, 1998). However, recent reports showed that GADD45 genes also function in cell survival (Gupta *et al*, 2006). PIM1 is a serine/threonine kinase that is involved in cell cycle progression and apoptosis (Bachmann and Moroy, 2005). DIDO1 was shown to be upregulated in early apoptosis and to trigger apoptosis (Garcia-Domingo *et al*, 2003). These data showed that the gene expression profile well reflects the functional pro-proliferative effects of anti-EpCAM antibody.

Several differentially expressed genes can be considered as p53 network, which might play a role in the signal transduction leading to the proliferative effects of EpCAM antibody treatment. p53 expression was downregulated upon EpCAM antibody treatment possibly due to an overexpression of its regulator Mdm2. Cyclin G (Ccng2) was one of the earliest p53 target genes. Moreover, cyclin G directly interacts with Mdm2 and can stimulate the ability of PP2A to dephosphorylate Mdm2, leading to a degradation of p53 (Chen, 2002). In contrast, the p53 activator Lats2 was found to be upregulated. However, induction of other genes inhibiting p53 and repression of genes positively regulating p53 might as well contribute to the downregulation of p53. The transcription factor Jun is known to inhibit p53 transcription, whereas the transcription factor Sp1 is known to be a positive regulator of p53. The RB1-inducible coiled-coil 1 (Rb1cc1) was shown to stabilise p53 (Melkoumian *et al*, 2005). Likewise, the phosphorylation of p53 by mitogen-activated protein kinase 14 (Mapk14), also known as p38, activates p53 (Harris and Levine, 2005). Epithelial cell adhesion molecule expression has recently been associated with cell cycle regulation. Ectopic expression of EpCAM in kidney cells resulted in the upregulation of c-myc as well as cyclins A and E (Munz *et al*, 2004). In this study, the expression of cyclin A was also found to be upregulated in A2C12 cells after 48 h of EpCAM treatment.

The application of EpCAM-specific antibodies as antineoplastic agents led to inconsistent results (reviewed in Baeuerle and Gires (2007) and Chaudry *et al* (2007)). The mechanisms by which anti-EpCAM antibodies exert tumour inhibition *in vivo* remain controversial. The cytotoxic mechanisms include antibody-dependent cell cytotoxicity mediated by natural killer cells and T lymphocytes, complement-mediated cytolysis, and opsonisation promoting phagocytosis mediated by polymorphonuclear cells. Taken together, EpCAM antibody treatment seems to make tumour cells recognisable for immune response *in vivo*, and the antineoplastic effects of the antibody require the immune response. These *in vivo* effects might overlay the possible EpCAM antibody-triggered pro-proliferative intracellular signalling seen in this study. However, future studies will have to show whether anti-EpCAM antibodies clinically applied as antitumour agents display the same pro-proliferative intracellular signalling as the antibody G8.8 used in this study.

## Figures and Tables

**Table 1:** Analysis of function of differentially expressed genes


	**Number of differentially expressed genes (induced/repressed)**					
**(A) Functional category**	**A2C12**	**A549**	**Caco-2**	**A2C12/A549^a^**		
Cell cycle^b^	232 (126/106)	153 (73/80)	33 (25/8)	29 (20/9)		
Cellular growth and proliferation^c^	415 (204/211)	306 (139/167)	46 (33/13)	48 (29/19)		
Cell death^d^	395 (202/193)	265 (128/137)	62 (48/14)	49 (33/16)		
Cancer^e^	462 (242/220)	344 (155/189)	77 (54/23)	54 (31/23)		
						

	**Number of differentially expressed genes (induced/repressed)**					
**(B) Cell cycle phase**	**A2C12**	**A549**	**Caco-2**	**A2C12/A549/Caco-2^a^**	**A2C12/A549^a^**	**A549/Caco-2^a^**
G0/G1 phases and G1/S transition	58 (25/33)	38 (20/18)	8 (5/3)	2 (2/0)	9 (6/3)	3 (2/1)
S phase	36 (21/15)	28 (16/12)	1 (0/1)	1 (1/0)	4 (3/0)	1 (0/1)
G2/M phases	38 (27/11)	18 (12/6)	5 (4/1)	3 (3/0)	4 (4/0)	0

(A) Most significantly over-represented functional categories in the group of significantly regulated genes are shown. (B) Analysis of cell cycle-related differentially expressed genes.

a Commonly modulated in the cell lines mentioned.

b Includes the functions and stages of the cell cycle including cell division. Functions associated with mitosis and meiosis are included in this category. Some examples of functions in this category are assembly of telomeres, cell cycle progression, and G0 phase of cells.

c Includes functions associated with the growth and proliferation of cells. Some examples of these functions include colony formation, proliferation, and outgrowth of cells.

d Includes functions associated with cellular death and survival. Some examples of functions included in this category are cytolysis, necrosis, survival, and recovery of cells.

e Includes functions associated with cancer. This includes any process associated with a tumour, cancer cell, or cancerous tissue, as well as any object associated with a cancer process such as transformation and metastasis. This category also includes all cancerous diseases.

**Table 2:** Genes differentially regulated by anti-EpCAM antibody in A2C12, A549, and Caco-2 cells in parallel

**Probe set ID**					**Fold change**		
**A2C12**	**A549**	**Caco-2**	**Gene symbol**	**Gene title**	**A2C12**	**A549**	**Caco-2**
1426156_at	230348_at	230348_at	LATS2	Large tumour suppressor homologue 2	16	9.9	4.3
1450971_at	209304_x_at	209304_x_at	GADD45B	Growth arrest and DNA damage-inducible gene 45*β*	18.4	2.5	2.3
1422931_at	218880_at	218880_at	FOSL2	FOS-like antigen 2	12.1	6.5	2.1
1435458_at	209193_at	209193_at	PIM1	Pim-1 oncogene	13.9	4.6	2.1
1456405_at	227335_at	227335_at	DIDO1	Death inducer-obliterator	−5.3	−3.5	−5.7
1438322_x_at	239358_at	239358_at	FDFT1	Farnesyl-diphosphate farnesyltransferase	2.1	13.9	2.0
1427285_s_at	226675_s_at	226675_s_at	MALAT1	Metastasis-associated lung adenocarcinoma transcript 1 (non-coding)	5.3	2.1	3.7
1454694_a_at	237469_at	237469_at	TOP2A	Topoisomerase II*α*	2.6	2.8	2.8
1416958_at	209750_at	209750_at	NR1D2	Nuclear receptor subfamily 1, group D, member 2	2.0	2.3	2.3
1455171_at	218242_s_at	222566_at	SUV420H1	Suppressor of variegation 4-20 homologue 1	−4.3	−2.6	−2.5
1424055_at	239815_at	225145_at	NCOA5	Nuclear receptor coactivator 5	−2.1	−3.7	−3.0
1423667_at	200769_s_at	200769_s_at	MAT2A	Methionine adenosyltransferase II*α*	−3.0	−2.1	−2.3
1460179_at	200880_at	200880_at	DNAJA1	DnaJ (Hsp40) homologue, subfamily A, member 1	−2.8	−2.0	−2.1

EpCAM=epithelial cell adhesion molecule.

**Table 3:** Modulation of gene expression as detected by microarray analysis and real-time PCR

	**A2C12**			**A549**		**Caco-2**	
	**Fold change**			**Fold change**		**Fold change**	
**Gene**	**Microarray analysis^a^**	**Real-time PCR**	**Gene**	**Microarray analysis^a^**	**Real-time PCR**	**Microarray analysis^a^**	**Real-time PCR**
Lats2	16.0	1.7	LATS2	9.9	2.5^b^	4.3	2.5^b^
Gadd45b	18.4	Induced^c^	GADD45B	2.5	Induced^c^	2.3	Induced^c^
Mdm2	2.1	1.5^b^	MDM2	2.9	3.2^b^	Nc^d^	1.3
Pim1	13.9	Induced^c^	PIM1	4.6	10.7^b^	2.1	2.9^b^
Trp53	−3.5	2.4^b^	TP53	Nc^d^	2.8^b^	Nc^d^	−1.5
Ccna2	2.6	2.4^b^	CCNA2	1.5	4.1^b^	Nc^d^	1.1
Ccnd2	−3.3	−3.2^b^	CCND2	Nc^d^	2.3	1.5	2.5^b^

EpCAM=epithelial cell adhesion molecule.

a In the case of more than one probe set identifying modulated gene expression, the highest fold change value is shown. All values ⩽−2 or ⩾2 are statistically significant for *P*<0.002. Fold changes between −2 and 2 have not been considered as significant according to the applied criteria (see Materials and Methods).

b Differences of gene expression between EpCAM antibody-treated and untreated cells were statistically significant (*P*<0.05).

c Fold change could not be calculated, as no expression was detectable in the non-treated control probe. Expression of the specific PCR product in the antibody-treated probes was shown by agarose gel electrophoresis (Supplementary Figure S1).

d Nc: no change.

## References

[bib1] Armstrong A, Eck SL (2003) EpCAM: a new therapeutic target for an old cancer antigen. Cancer Biol Ther 2: 320–326 14508099 10.4161/cbt.2.4.451

[bib2] Aylon Y, Michael D, Shmueli A, Yabuta N, Nojima H, Oren M (2006) A positive feedback loop between the p53 and Lats2 tumor suppressors prevents tetraploidization. Genes Dev 20: 2687–2700 17015431 10.1101/gad.1447006 PMC1578695

[bib3] Bachmann M, Moroy T (2005) The serine/threonine kinase Pim-1. Int J Biochem Cell Biol 37: 726–730 15694833 10.1016/j.biocel.2004.11.005

[bib4] Baeuerle PA, Gires O (2007) EpCAM (CD326) finding its role in cancer. Br J Cancer 96: 417–423 17211480 10.1038/sj.bjc.6603494 PMC2360029

[bib5] Borlak J, Meier T, Halter R, Spanel R, Spanel-Borowski K (2005) Epidermal growth factor-induced hepatocellular carcinoma: gene expression profiles in precursor lesions, early stage and solitary tumours. Oncogene 24: 1809–1819 15674348 10.1038/sj.onc.1208196

[bib6] Chaudry MA, Sales K, Ruf P, Lindhofer H, Winslet MC (2007) EpCAM an immunotherapeutic target for gastrointestinal malignancy: current experience and future challenges. Br J Cancer 96: 1013–1019 17325709 10.1038/sj.bjc.6603505 PMC2360124

[bib7] Chen X (2002) Cyclin G: a regulator of the p53-Mdm2 network. Dev Cell 2: 518–519 12015958 10.1016/s1534-5807(02)00182-x

[bib8] Fagerberg J, Hjelm AL, Ragnhammar P, Frodin JE, Wigzell H, Mellstedt H (1995) Tumor regression in monoclonal antibody-treated patients correlates with the presence of anti-idiotype-reactive T lymphocytes. Cancer Res 55: 1824–1827 7728746

[bib9] Garcia-Domingo D, Ramirez D, Gonzalez dB, Martinez A (2003) Death inducer-obliterator 1 triggers apoptosis after nuclear translocation and caspase upregulation. Mol Cell Biol 23: 3216–3225 12697821 10.1128/MCB.23.9.3216-3225.2003 PMC153187

[bib10] Gastl G, Spizzo G, Obrist P, Dunser M, Mikuz G (2000) Ep-CAM overexpression in breast cancer as a predictor of survival. Lancet 356: 1981–1982 11130529 10.1016/S0140-6736(00)03312-2

[bib11] Guillemot JC, Naspetti M, Malergue F, Montcourrier P, Galland F, Naquet P (2001) Ep-CAM transfection in thymic epithelial cell lines triggers the formation of dynamic actin-rich protrusions involved in the organization of epithelial cell layers. Histochem Cell Biol 116: 371–378 11702195 10.1007/s004180100329

[bib12] Gupta M, Gupta SK, Hoffman B, Liebermann DA (2006) Gadd45a and Gadd45b protect hematopoietic cells from UV-induced apoptosis via distinct signaling pathways, including p38 activation and JNK inhibition. J Biol Chem 281: 17552–17558 16636063 10.1074/jbc.M600950200

[bib13] Hansen T, Blickwede M, Borlak J (2006) Primary rat alveolar epithelial cells for use in biotransformation and toxicity studies. Toxicol *In vitro* 20: 757–766 16326067 10.1016/j.tiv.2005.10.011

[bib14] Harris SL, Levine AJ (2005) The p53 pathway: positive and negative feedback loops. Oncogene 24: 2899–2908 15838523 10.1038/sj.onc.1208615

[bib15] Herlyn M, Steplewski Z, Herlyn D, Koprowski H (1979) Colorectal carcinoma-specific antigen: detection by means of monoclonal antibodies. Proc Natl Acad Sci USA 76: 1438–1442 286328 10.1073/pnas.76.3.1438 PMC383267

[bib16] Kim JW, Ye Q, Forgues M, Chen Y, Budhu A, Sime J, Hofseth LJ, Kaul R, Wang XW (2004) Cancer-associated molecular signature in the tissue samples of patients with cirrhosis. Hepatology 39: 518–527 14768006 10.1002/hep.20053

[bib17] Koprowski H, Steplewski Z, Mitchell K, Herlyn M, Herlyn D, Fuhrer P (1979) Colorectal carcinoma antigens detected by hybridoma antibodies. Somatic Cell Genet 5: 957–971 94699 10.1007/BF01542654

[bib18] Kuhn S, Koch M, Nubel T, Ladwein M, Antolovic D, Klingbeil P, Hildebrand D, Moldenhauer G, Langbein L, Franke WW, Weitz J, Zoller M (2007) A complex of EpCAM, claudin-7, CD44 variant isoforms, and tetraspanins promotes colorectal cancer progression. Mol Cancer Res 5: 553–567 17579117 10.1158/1541-7786.MCR-06-0384

[bib19] Maaser K, Höpfner M, Jansen A, Weisinger G, Gavish M, Kozikowski AP, Weizman A, Carayon P, Riecken EO, Zeitz M, Scherübl H (2001) Specific ligands of the peripheral benzodiazepine receptor induce apoptosis and cell cycle arrest in human colorectal cancer cells. Br J Cancer 85: 1771–1780 11742501 10.1054/bjoc.2001.2181 PMC2363981

[bib20] Melkoumian ZK, Peng X, Gan B, Wu X, Guan JL (2005) Mechanism of cell cycle regulation by FIP200 in human breast cancer cells. Cancer Res 65: 6676–6684 16061648 10.1158/0008-5472.CAN-04-4142

[bib21] Munz M, Kieu C, Mack B, Schmitt B, Zeidler R, Gires O (2004) The carcinoma-associated antigen EpCAM upregulates c-myc and induces cell proliferation. Oncogene 23: 5748–5758 15195135 10.1038/sj.onc.1207610

[bib22] Naundorf S, Preithner S, Mayer P, Lippold S, Wolf A, Hanakam F, Fichtner I, Kufer P, Raum T, Riethmuller G, Baeuerle PA, Dreier T (2002) *In vitro* and *in vivo* activity of MT201, a fully human monoclonal antibody for pancarcinoma treatment. Int J Cancer 100: 101–110 12115595 10.1002/ijc.10443

[bib23] Osta WA, Chen Y, Mikhitarian K, Mitas M, Salem M, Hannun YA, Cole DJ, Gillanders WE (2004) EpCAM is overexpressed in breast cancer and is a potential target for breast cancer gene therapy. Cancer Res 64: 5818–5824 15313925 10.1158/0008-5472.CAN-04-0754

[bib24] Punt CJ, Nagy A, Douillard JY, Figer A, Skovsgaard T, Monson J, Barone C, Fountzilas G, Riess H, Moylan E, Jones D, Dethling J, Colman J, Coward L, MacGregor S (2002) Edrecolomab alone or in combination with fluorouracil and folinic acid in the adjuvant treatment of stage III colon cancer: a randomised study. Lancet 360: 671–677 12241873 10.1016/S0140-6736(02)09836-7

[bib25] Riethmuller G, Holz E, Schlimok G, Schmiegel W, Raab R, Hoffken K, Gruber R, Funke I, Pichlmaier H, Hirche H, Buggisch P, Witte J, Pichlmayr R (1998) Monoclonal antibody therapy for resected Dukes' C colorectal cancer: seven-year outcome of a multicenter randomized trial. J Clin Oncol 16: 1788–1794 9586892 10.1200/JCO.1998.16.5.1788

[bib26] Riethmuller G, Schneider-Gadicke E, Schlimok G, Schmiegel W, Raab R, Hoffken K, Gruber R, Pichlmaier H, Hirche H, Pichlmayr R (1994) Randomised trial of monoclonal antibody for adjuvant therapy of resected Dukes' C colorectal carcinoma. German Cancer Aid 17-1A Study Group. Lancet 343: 1177–1183 7909866 10.1016/s0140-6736(94)92398-1

[bib27] Schiechl H, Dohr G (1987) Immunohistochemical studies of the distribution of a basolateral-membrane protein in intestinal epithelial cells (GZ1-Ag) in rats using monoclonal antibodies. Histochemistry 87: 491–498 3323146 10.1007/BF00496823

[bib28] Schon MP, Schon M, Klein CE, Blume U, Bisson S, Orfanos CE (1994) Carcinoma-associated 38-kD membrane glycoprotein MH 99/KS 1/4 is related to proliferation and age of transformed epithelial cell lines. J Invest Dermatol 102: 987–991 8006466 10.1111/1523-1747.ep12384258

[bib29] Seligson DB, Pantuck AJ, Liu X, Huang Y, Horvath S, Bui MH, Han KR, Correa AJ, Eeva M, Tze S, Belldegrun AS, Figlin RA (2004) Epithelial cell adhesion molecule (KSA) expression: pathobiology and its role as an independent predictor of survival in renal cell carcinoma. Clin Cancer Res 10: 2659–2669 15102668 10.1158/1078-0432.ccr-1132-03

[bib30] Songun I, Litvinov SV, van de Velde CJ, Pals ST, Hermans J, van Krieken JH (2005) Loss of Ep-CAM (CO17-1A) expression predicts survival in patients with gastric cancer. Br J Cancer 92: 1767–1772 15870832 10.1038/sj.bjc.6602519 PMC2362035

[bib31] Spizzo G, Obrist P, Ensinger C, Theurl I, Dunser M, Ramoni A, Gunsilius E, Eibl G, Mikuz G, Gastl G (2002) Prognostic significance of Ep-CAM AND Her-2/neu overexpression in invasive breast cancer. Int J Cancer 98: 883–888 11948467 10.1002/ijc.10270

[bib32] Spizzo G, Went P, Dirnhofer S, Obrist P, Moch H, Baeuerle PA, Mueller-Holzner E, Marth C, Gastl G, Zeimet AG (2006) Overexpression of epithelial cell adhesion molecule (Ep-CAM) is an independent prognostic marker for reduced survival of patients with epithelial ovarian cancer. Gynecol Oncol 103: 483–488 16678891 10.1016/j.ygyno.2006.03.035

[bib33] Spizzo G, Went P, Dirnhofer S, Obrist P, Simon R, Spichtin H, Maurer R, Metzger U, von Castelberg B, Bart R, Stopatschinskaya S, Kochli OR, Haas P, Mross F, Zuber M, Dietrich H, Bischoff S, Mirlacher M, Sauter G, Gastl G (2004) High Ep-CAM expression is associated with poor prognosis in node-positive breast cancer. Breast Cancer Res Treat 86: 207–213 15567937 10.1023/B:BREA.0000036787.59816.01

[bib34] Stoecklein NH, Siegmund A, Scheunemann P, Luebke AM, Erbersdobler A, Verde PE, Eisenberger CF, Peiper M, Rehders A, Esch JS, Knoefel WT, Hosch SB (2006) Ep-CAM expression in squamous cell carcinoma of the esophagus: a potential therapeutic target and prognostic marker. BMC Cancer 6: 165 16796747 10.1186/1471-2407-6-165 PMC1523209

[bib35] Takekawa M, Saito H (1998) A family of stress-inducible GADD45-like proteins mediate activation of the stress-responsive MTK1/MEKK4 MAPKKK. Cell 95: 521–530 9827804 10.1016/s0092-8674(00)81619-0

[bib36] Tulchinsky E (2000) Fos family members: regulation, structure and role in oncogenic transformation. Histol Histopathol 15: 921–928 10963134 10.14670/HH-15.921

[bib37] Varga M, Obrist P, Schneeberger S, Muhlmann G, Felgel-Farnholz C, Fong D, Zitt M, Brunhuber T, Schafer G, Gastl G, Spizzo G (2004) Overexpression of epithelial cell adhesion molecule antigen in gallbladder carcinoma is an independent marker for poor survival. Clin Cancer Res 10: 3131–3136 15131054 10.1158/1078-0432.ccr-03-0528

[bib38] Went P, Vasei M, Bubendorf L, Terracciano L, Tornillo L, Riede U, Kononen J, Simon R, Sauter G, Baeuerle PA (2006) Frequent high-level expression of the immunotherapeutic target Ep-CAM in colon, stomach, prostate and lung cancers. Br J Cancer 94: 128–135 16404366 10.1038/sj.bjc.6602924 PMC2361083

[bib39] Winter MJ, Cirulli V, Briaire-de Bruijn IH, Litvinov SV (2007) Cadherins are regulated by Ep-CAM via phosphaditylinositol-3 kinase. Mol Cell Biochem 302: 19–26 17646933 10.1007/s11010-007-9420-y

[bib40] Winter MJ, Nagelkerken B, Mertens AE, Rees-Bakker HA, Briaire-de Bruijn IH, Litvinov SV (2003a) Expression of Ep-CAM shifts the state of cadherin-mediated adhesions from strong to weak. Exp Cell Res 285: 50–58 12681286 10.1016/s0014-4827(02)00045-9

[bib41] Winter MJ, Nagtegaal ID, van Krieken JH, Litvinov SV (2003b) The epithelial cell adhesion molecule (Ep-CAM) as a morphoregulatory molecule is a tool in surgical pathology. Am J Pathol 163: 2139–2148 14633587 10.1016/S0002-9440(10)63570-5 PMC1892395

[bib42] Wurfel J, Rosel M, Seiter S, Claas C, Herlevsen M, Weth R, Zoller M (1999) Metastasis-association of the rat ortholog of the human epithelial glycoprotein antigen EGP314. Oncogene 18: 2323–2334 10327052 10.1038/sj.onc.1202542

[bib43] Yamashita A, Hakura A, Inoue H (1999) Suppression of anchorage-independent growth of human cancer cell lines by the drs gene. Oncogene 18: 4777–4787 10490811 10.1038/sj.onc.1202852

[bib44] Yamashita T, Budhu A, Forgues M, Wang XW (2007) Activation of hepatic stem cell marker EpCAM by Wnt-beta-catenin signaling in hepatocellular carcinoma. Cancer Res 67: 10831–10839 18006828 10.1158/0008-5472.CAN-07-0908

