# Supplementary material for: A genome-wide expression analysis identifies a network of EpCAM-induced cell cycle regulators
Source: Br J Cancer. 2008 Oct 28;99(10):1635–43. doi: 10.1038/sj.bjc.6604725 (PMC2584962; doi:10.1038/sj.bjc.6604725)
Supplement: Supplementary Figure S1 [file 6604725x1.ppt]

## Slide 1
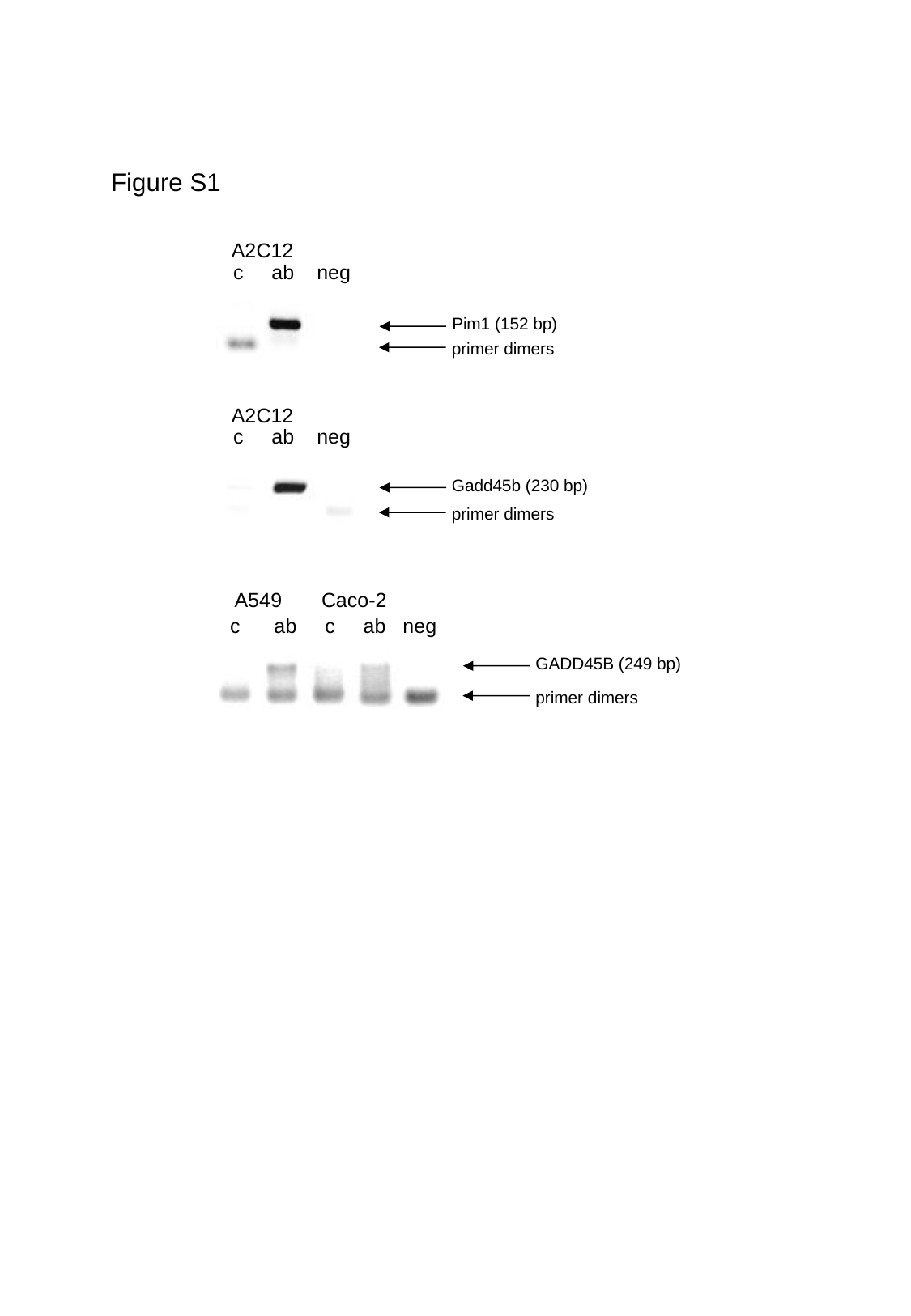

Figure S1
A2C12
c ab neg
Pim1 (152 bp)
primer dimers
A2C12
c ab neg
Gadd45b (230 bp)
primer dimers
A549 Caco-2
c ab c ab neg
GADD45B (249 bp)
primer dimers
