# Supplementary material for: A genome-wide expression analysis identifies a network of EpCAM-induced cell cycle regulators
Source: Br J Cancer. 2008 Oct 28;99(10):1635–43. doi: 10.1038/sj.bjc.6604725 (PMC2584962; doi:10.1038/sj.bjc.6604725)
Supplement: Supplementary Tables S1 and S2 [file 6604725x2.doc]

**Table S1: Real-time PCR primer sequences and amplification settings**

| Gene | Primer sequence | Fragment length (bp) | Primer annealing (°C) | Elon-gation (sec) | Denatu-ration^1^  (sec) | Tm ^2^  (°C) |
| --- | --- | --- | --- | --- | --- | --- |
| Human | | | | | | |
| LATS2 | fwd: TTCATCCACCGAGACATCAA  rev: CTCCATGCTGTCCTGTCTGA | 153 | 65 | 7 | 90/1 | 84 |
| GADD45B | fwd: GTGTACGAGTCGGCCAAGTT  rev: GACCAGGAGACAATGCAGGT | 249 | 60 | 10 | 90/1 | 81 |
| MDM2 | fwd: GAAGGAAACTGGGGAGTCTTG  rev: GGTCTCTTGTTCCGAAGCTG | 150 | 65 | 7 | 90/1 | 84 |
| PIM1 | fwd: CGAGCATGACGAAGAGATCA  rev: CCAGAAAGGCTGCTATTTGC | 229 | 65 | 10 | 90/1 | 81 |
| TP53 | fwd: GTGGAAGGAAATTTGCGTGT  rev: CCAGTGTGATGATGGTGAGG | 184 | 68 | 8 | 90/1 | 81 |
| CCNA2 | fwd: TTATTGCTGGAGCTGCCTTT  rev: CTCTGGTGGGTTGAGGAGAG | 224 | 65 | 10 | 90/1 | 81 |
| CCND2 | fwd: TGGGGAAGTTGAAGTGGAAC  rev: ATCATCGACGGTGGGTACAT | 175 | 60 | 7 | 120/0 | 83 |
| Gapdh | fwd: ggccaaggtcatccatga  rev: tcagtgtagcccaggatg | 353 | 68 | 15 | 120/0 | 88 |
| MTATP6 | fwd: ctaaaggacgaacctga  rev: tggcctgcagtaatgtt | 315 | 55 | 13 | 120/0 | 83 |
| Mouse | | | | | | |
| Lats2 | fwd: GTGTCCACAAGATGGGCTTT  rev: CTCCATGCTGTCCTGTCTCA | 170 | 65 | 7 | 90/1 | 81 |
| Gadd45b | fwd: CACCCTGATCCAGTCGTTCT  rev: CCCATTGGTTATTGCCTCTG | 230 | 65 | 10 | 90/1 | 81 |
| Mdm2 | fwd: TGCAAGCACCTCACAGATTC  rev: ACACAATGTGCTGCTGCTTC | 188 | 68 | 8 | 90/1 | 81 |
| Pim1 | fwd: CTTCGGCTCGGTCTACTCTG  rev: CCGAGCTCACCTTCTTCAAC | 152 | 65 | 7 | 90/1 | 84 |
| Trp53 | fwd: AGAGACCGCCGTACAGAAGA  rev: CTGTAGCATGGGCATCCTTT | 232 | 65 | 10 | 90/1 | 84 |
| Ccna2 | fwd: ACCTGCCTTCACTCATTGCT  rev: TTGACTGTTGGGCATGTTGT | 177 | 68 | 8 | 90/1 | 81 |
| Ccnd2 | fwd: TTACCTGGACCGTTTCTTGG  rev: TGCTCAATGAAGTCGTGAGG | 240 | 65 | 10 | 90/1 | 84 |
| Gapdh | fwd: ggccaaggtcatccatga  rev: tcagtgtagcccaggatg | 353 | 68 | 15 | 120/0 | 88 |
| Mtatp6 | fwd: ctaaaggacgaacctga  rev: tggcctgcagtaatgtt | 315 | 55 | 13 | 120/0 | 83 |

^1^ Denaturation time (sec): 90 or 120 sec at 95 °C as a first step before PCR and 0 or 1 sec at 95 °C within each PCR cycle.

^2^ Fluorescence measurement temperature.

**Table S2: Number of differentially expressed probe sets**

|  | Differentially expressed gene transcripts | | |
| --- | --- | --- | --- |
|  | Total | Increased | Decreased |
| A2C12 | 1973 | 901 | 1072 |
| A549 | 1743 | 589 | 1154 |
| Caco-2 | 378 | 246 | 132 |
| A2C12/A549/Caco-2 | 13 | 8 | 5 |
