# Supplementary material for: A genome-wide expression analysis identifies a network of EpCAM-induced cell cycle regulators
Source: Br J Cancer. 2008 Oct 28;99(10):1635–43. doi: 10.1038/sj.bjc.6604725 (PMC2584962; doi:10.1038/sj.bjc.6604725)
Supplement: Supplementary Figure Legend [file 6604725x3.doc]

**Figure legends**

**Figure S1: Expression of Pim-1 and Gadd45b was induced by anti-EpCAM treatment.**

Specificity of real-time PCR products was shown by agarose gel electrophoresis. A distinct expression of Pim-1 and Gadd45b was shown in EpCAM-antibody treated A2C12 cells (ab), whereas in non-treated cells (c) no expression of Pim-1 or Gadd45b was detected. Likewise, GADD45B was only detectable in EpCAM-antibody treated (ab) A549 and Caco-2 cells, but not in non-treated (c) cells. Negative control PCR (neg) was conducted by omitting c-DNA.
